# METTL3 promotes non-small cell lung cancer (NSCLC) cell proliferation and colony formation in a m6A-YTHDF1 dependent way

**DOI:** 10.1186/s12890-022-02119-3

**Published:** 2022-08-25

**Authors:** Xuejun Dou, Zhiyuan Wang, Weiqiang Lu, Libin Miao, Yuefeng Zhao

**Affiliations:** Department of Thoracic Surgery, Space Central Hospital, Beijing, China

**Keywords:** METTL3, Non-small cell lung cancer (NSCLC), Cell proliferation, Colony formation, YTHDF1, FRAS1, CDON

## Abstract

**Background:**

N6-methyladenosine (m6A) is the most common RNA modification, which plays a pivotal role in tumor development and progression. In this study, we assessed the role of m6A methyltransferase METTL3 in FRAS1-involved cell proliferation and colony formation of non-small cell lung cancer (NSCLC) cell lines.

**Methods:**

Cell viability was analyzed by Cell Counting Kit (CCK-8) and colony formation. M6A RNA immunoprecipitation (IP), Ribosomal immunoprecipitation, RNA immunoprecipitation (RIP) were performed to verify the relationship between METTL3, FRAS1 and YTHDF1. Rescue experiments to confirm the regulatory mechanism of METTL3-FRAS1 promoted NSCLC cell proliferation through CDON by cooperating YTHDF1.

**Results:**

We found that FRAS1 was correlated with poor prognosis in NSCLC patients, of which the transcript undergoes m6A modification regulated by METTL3. METTL3 silence reduced cell viability of NSCLC cells HCC827 and NCI-H1975, which could be restored by FRAS1 overexpression. The m6A modification of FRAS1 could be recognized by YTHDF1. FRAS1 silence or YTHDF1 silence could rescue the elevated NSCLC cell proliferation, colony formation, and tumor growth induced by METTL3 overexpression in vitro and in vivo.

**Conclusions:**

Our study reveals that METTL3-FRAS1 plays a crucial role in NSCLC cell proliferation, colony formation, and tumor growth through the regulation of CDON by cooperating YTHDF1.

**Supplementary Information:**

The online version contains supplementary material available at 10.1186/s12890-022-02119-3.

## Introduction

N6-methyladenosine (m6A) modification has been identified to play a vital role in tumorigenesis [1–3]. Abnormal m6A modifications have been reported to be associated with glioblastoma (GBM), ovarian cancer, bladder cancer, gastric cancer, pancreatic cancer and so on [4–6]. Existing studies showed that m6A modification acts the dual roles in cancers. Nonetheless, there are still largely unknown about the role, the mechanism and the clinical value of m6A in lung cancer.

M6A modification is regulated by specific methyltransferases (referred to as “writers”), m6A recognition proteins (referred to as “readers”) and demethylases (referred to as “erasers”). METTL3 is the first identified methyltransferase which is responsible for m6A modification. Emerging evidence have shown that METTL3 plays multiple functions in tumorigenesis [7, 8]. For instance, METTL3 promotes liver cancer progression via regulating SOCS2 expression in an m6A-dependent manner [9]. In gastric cancer (GC), METTL3 promotes cell proliferation and liver metastasis in GC by enhancing m6A modification and stability of *HDGF* [10]. It is reported that METTL3 accelerates colorectal cancer progression by inducing m6A modification of *GLUT1* and activating mTORC1 signaling [11]. METTL3 is also found to facilitate breast cancer tumorigenesis by regulating Bcl-2 [12]. Recent research reveals that METTL3 is upregulated in lung cancer [13]. METTL3-induced m6A methylation of lncRNA ABHD11-AS1 could promote cell proliferation and Warburg effect of NSCLC [14]. These studies indicate that METTL3-modulated m6A mRNA methylation plays vital roles in human tumors including lung cancer.

YT521-B homology (YTH) domain-containing proteins (YTHDFs) YTH could recognize and regulate the effects of RNA m6A modification. YTHDF2 is the first reported m6A reader and studies have shown that YTHDF2 promotes tumorigenesis in multiple cancers [2]. For example, YTHDF2 could promote glioblastoma growth by remaining the stability of *MYC* and *VEGFA* transcripts in an m6A-dependent way [15]. In hepatocellular carcinoma (HCC), one research shows that YTHDF2 expression has a positive correlation with HCC progression [16] whereas another research reports that YTHDF2 suppressed HCC tumor growth through accelerating *EGFR* mRNA degradation [17]. Except for YTHDF2, the YTHDFs family contains YTHDF1, YTHDF3, YTHDC1. It is reported that YTHDF1 functions as a tumor promoter in various cancers such as gastric cancer [18], HCC [19, 20], NSCLC [21]. Up to now, little is known about the role of YTHDF1 in NSCLC.

Fraser extracellular matrix complex subunit 1 (FRAS1) has been reported to be essential for embryonic epithelial-mesenchymal integrity [22]. Recent studies show that FRAS1 contributes to the malignant phenotype of NSCLC [23], gastric cancer [24], colorectal cancer [25].

In this study, we explored the role and the potential molecular mechanism of FRAS1 in NSCLC. Our data demonstrates that m6A methyltransferase METTL3 induced *FRAS1* m6A modification and protein stability, thus accelerating NSCLC cell proliferation.

## Methods

### Data analysis

The overall survival analysis of FRAS1 in non-small-cell lung cancer (NSCLC) was assessed by using the online survival analysis tool Kaplan–Meier plotter (https://kmplot.com/analysis/index.php?p=background) [26], containing the clinical information of NSCLC patients (https://kmplot.com/analysis/index.php?p=service&cancer=lung) [27]. The NSCLC patients were divided into two groups by the medium expression of FRAS1. The hazard ratio (HR) with 95% confidence intervals (CIs) and log rank p value were labeled in the curve. The JetSet probe set for FRAS1 (220910_at) was used for analysis. Pearson correlation analysis was performed to identify co-expressed genes related to METTL3 and YTHDF1 with a cut-off of the |Pearson R|> 0.3 and *P* < 0.05 in TCGA-LUAD&LUSC dataset. According to the correlation coefficient for METTL3 greater than 0.6, correlation coefficient for YTHDF1 greater than 0.45, and P value less than 0.001, the intersection genes were screened out and listed in the Additional file 1: Table S1.

### Human tissues and cell lines

This study includes five pairs of lung adenocarcinoma (LUAD) tissues from Space Central Hospital. The ethical approval for this study was from Space Central Hospital (20,200,511-JJJHZ-01). Written informed consents were obtained from each patient. Four NSCLC cell lines (A549, NCI-H1299, HCC827, and NCI-H1975) and human bronchial epithelial cell line HBEC were obtained from Procell Life Science&Technology Co.,Ltd (Wuhan, China). A549 cells were cultured in DMEM (High glucose, Gibco, Grand Island, NY, USA, 11,965–092) with 10% FBS (Gibco) and 1% penicillin/streptomycin (P/S, Procell). NCI-H1299, HCC827, and NCI-H1975 cells were cultured in RPMI-1640 medium (Gibco) with 10% FBS (Gibco) and 1% penicillin/streptomycin (P/S, Procell). HBEC cells were cultured in the bronchial epithelial growth medium (BEGM, Lonza, CC-3170). All the cells were cultured at 37 °C in a 5% CO_2_ incubator.

### Stable cell lines

shMETTL3, shYTHDF1, shFRAS1 and shScramble were synthesized from RiboBio (Guangzhou, China), which were cloned into the vector pLKO.1 puro. The stable cell lines such as shMETTL3, shYTHDF1, shFRAS1 and shscramble were generated by infecting these corresponding lentivirus. Two days after infection, the puromycin (1 μg/mL) was added. The shRNA sequences were shown in Table 1.

**Table 1:** Sequences of shRNAs

Target	Oligos
shMETTL3	5′-ACCTCGCTGCACTTCAGACGAATTATTCAAGAGATAATTCGTCTGAAGTGCAGCTT-3′
	5′-CAAAAAGCTGCACTTCAGACGAATTATCTCTTGAATAATTCGTCTGAAGTGCAGCG-3′
shYTHDF1	5′-ACCTCGCTGGAGAATAACGACAACAATCAAGAGTTGTTGTCGTTATTCTCCAGCTT-3′
	5′-CAAAAAGCTGGAGAATAACGACAACAACTCTTGATTGTTGTCGTTATTCTCCAGCG-3′
shFRAS1	5′-ACCTCGCTAGTGAAGTTAAACGTATTTCAAGAGAATACGTTTAACTTCACTAGCTT-3′
	5′-CAAAAAGCTAGTGAAGTTAAACGTATTCTCTTGAAATACGTTTAACTTCACTAGCG-3′
shscramble	5′-ACCTCGCGCATCGATTGCATACTATATCAAGAGTATAGTATGCAATCGATGCGCTT-3′
	5′-CAAAAAGCGCATCGATTGCATACTATACTCTTGATATAGTATGCAATCGATGCGCG-3′

### Quantitative Reverse Transcription-Polymerase chain reaction (qRT-PCR)

Total RNA was isolated using Trizol (Beyotime, Shanghai, China) and reverse transcription was performed with the EasyScript® First-Strand cDNA Synthesis SuperMix (Transgen, Beijing, China). Quantitative real-time PCR analysis was performed with the TransStart® Green qPCR SuperMix (Transgen). The relative expression of each gene was analyzed by the standard 2^−ΔΔCt^ method [28] and normalized to GAPDH. The sequences of qPCR Primers were listed in Table 2.

**Table 2:** Primers used for quantitative polymerase chain reaction

Gene	Forward primer (5′–3′)	Reverse primer (5′–3′)
FRAS1	CTAGCGTTGGCGGAATTTGC	GCATTGGTTGGCAGCTATTTGA
METTL3	TTGTCTCCAACCTTCCGTAGT	CCAGATCAGAGAGGTGGTGTAG
YTHDF1	ACCTGTCCAGCTATTACCCG	TGGTGAGGTATGGAATCGGAG
CDON	CAGAAACTTGGTGGACCTGTAG	GTTATGCAGCCATGAGATACGA
CCND1	GCTGCGAAGTGGAAACCATC	CCTCCTTCTGCACACATTTGAA
GAPDH	ATCATCCCTGCCTCTACTGG	GTCAGGTCCACCACTGACAC

### Western blot

Cells were lysed and proteins were extracted using the ice-cold lysis buffer. The equal amount of proteins were separated by 6% or 10% SDS-PAGE and transferred to nitrocellulose membrane. After blocked with 5% non-fat milk for 1 h at room temperature, membranes were incubated with corresponding primary antibodies at 4 °C overnight. Then the membranes were incubated with horseradish peroxidase (HRP)-conjugated secondary antibodies and sensed by the enhanced chemiluminescence (ECL) substrates (Beyotime, Shanghai, China). Primary antibodies were as follows: anti-FRAS1 (#ab240583; Abcam, Cambridge, UK), anti-METTL3 (#ab195352; Abcam), anti-YTHDF1 (#ab252346; Abcam), anti-RPL-22 (#ab77720; Abcam), anti-CDON (#ab227056; Abcam), anti-CCND1 (#ab16663; Abcam), anti-β-actin (#ab8226; Abcam), anti-α-tubulin (#ab7291, Abcam) and anti-GAPDH (#ab8245; Abcam).

### CCK-8 and colony formation

Cell viability was analyzed using the cell counting kit-8 (CCK-8, Beyotime). Cells were seeded in the 96-well plate (2 × 10^3^/well) and cultured for different time (0, 1, 2, 3, 4 or 5 days). Cells were incubated with 10 μL CCK-8 solution for 1 h. The absorbance at 450 nm was detected using a multimode plate reader.

For colony formation assay, cells were plated at a density of 4 × 10^3^/well in the 6-well plate with complete medium. After 2 weeks, cells were fixed with paraformaldehyde and then stained with 0.1% crystal violet. The colonies images were determined by using a camera.

### EDU incorporation

The chamber slides were coated with poly-D-lysine and matrigel. HCC827 and NCI-H1975 cells were seeded on the coated slides. The next day, cells were transfected with si-CDON or si-NC plasmids. After transfection for 48 h, EDU was added for 4 h before fixation. Cells were incubated with fixative solution for 15 min and permeabilized for 20 min. Then the reaction mix was used to fluorescently label EDU for 30 min. Cells were stained for DAPI for 30 min. After that, the EDU positive cells were analyzed using fluorescence microscope, ImageJ software and GraphPad Prism 8.0. The EDU incorporation was performed using Edu staining proliferation kit (Abcam, ab222421).

### RNA immunoprecipitation (RIP)

RIP was performed with EZ-Magna RIP RNA-Binding Protein Immunoprecipitation Kit (Millipore, Billerica, MA). Cells were lysed with specially formulated RIP lysis buffer. Cell lysates were incubated with the indicated antibodies together with the mixed protein A/G-beads at 4 °C overnight. The precipitated RNA was examined by qRT-PCR.

### RNA stability assays

HCC827 or NCI-H1975 cells were treated with actinomycin D (5 μg/mL, Sigma) for 0, 2, 4, 6 h. Total RNA was extracted by TRIzol and analyzed by qRT-PCR.

### m6A RNA immunoprecipitation assay (MeRIP)

Total RNA was isolated using TRIzol (Beyotime) and then sonicated into RNA fragments. Then, the RNA fragments were incubated with the antibody against m6A (ab208577, Abcam) together with protein A beads at 4 °C for 10 h. The protein-RNA complex was washed and treated with proteinase K. The immunoprecipitated RNA was detected by qRT-PCR.

### Ribosomal immunoprecipitation

HCC827 or NCI-H1975 cells with stable shYTHDF1 or shscr were transfected with RPL22-Myc (Origene, Beijing, China). For 48 h later, cells were lysed using RIPA buffer and then incubated with anti-Myc antibody (Abcam) mixed with protein A beads at 4 °C for 8 h. The normal mouse immunoglobulin G (IgG) was used as control. The protein-RNA complex was washed and treated with proteinase K. The immunoprecipitated RNA was detected by qRT-PCR.

### Xenograft tumor models

Twenty BALB/c nude mice (6–8 weeks old) were purchased from Beijing Viewsolid Biotechnology Co. LTD (Beijing, China). Mice were randomly divided into four groups (five mice/group). The stable cells of NCI-H1975/Vector + sh-NC, NCI-H1975/METTL3 + sh-NC, NCI-H1975/METTL3 + sh-YTHDF1, and NCI-H1975/METTL3 + sh-FRAS1 were infected and constructed by using lentivirus (Gene Pharma, Shanghai, China). The xenografts in each group were established by using subcutaneous injection with approximately 1 × 10^6^ cells in each mouse. Tumor volume was measured every 7 days. The tumor volume was calculated as 1/ 2 × length × width^2^. The tumor weight was recorded 28 days after inoculation.

### Statistical analysis

All experiments were independently repeated at least three times. Data was analyzed using GraphPad Prism 8.0 (GraphPad Software, La Jolla, CA). Group differences were analyzed using Student's t-test. The correlation between FRAS1 and CDON expression was tested via starbase RNA-RNA module (https://starbase.sysu.edu.cn/index.php). *P* < 0.05 was considered to be statistical significance.

## Results

### N6-methyladenosine (m6A) modification and protein level of *FRAS1* is upregulated in NSCLC

**Fig. 1:**
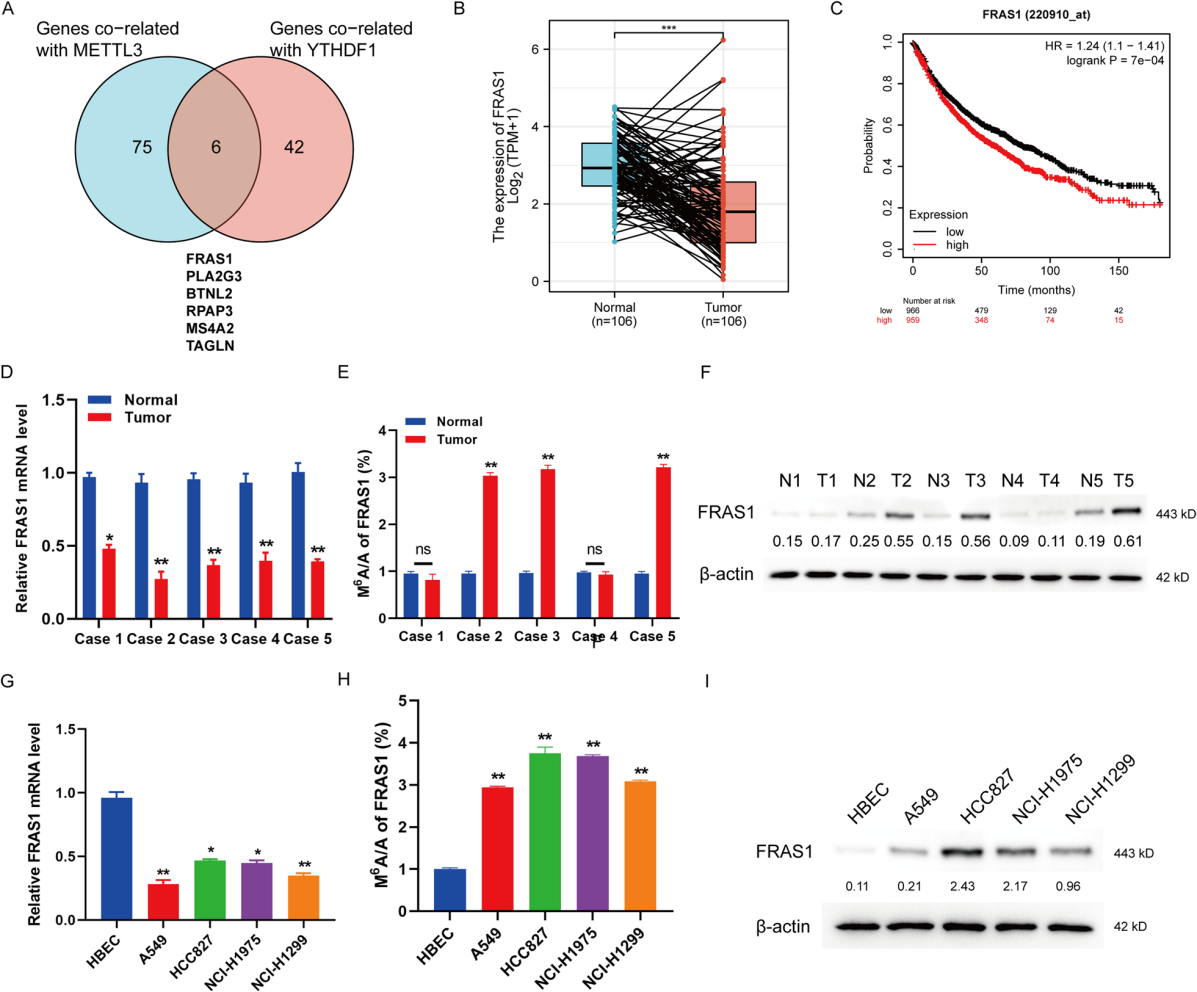
N6-methyladenosine modification and the protein expression of FRAS1 was increased in NSCLC tumor tissues and cell lines. **A** Venn diagram showed the intersection of the co-related genes with METTL3 and the co-related genes with YTHDF1 in lung cancer. There are six genes in the intersection list. FRAS1, PLA2G3, BTNL2, RPAP3, MS4A2, and TAGLN. **B** The mRNA level of FRAS1 was analyzed in paired NSCLC tumors tissues and normal tissues (n = 106) via TCGA. **C** The overall survival of NSCLC patients with FRAS1 expression was analyzed by using Kaplan–Meier plotter. Hazard ratio (HR) = 1.24, 95% Confidence Interval (CI) = 1.1–1.41, log rank *P* = 7e-04. **D** FRAS1 expression at mRNA level was analyzed by qRT-PCR in five pairs of LUAD samples. N: Normal tissues. T: Tumor tissues. **E** The m6A level of FRAS1 was determined by using MeRIP-qPCR in five pairs of LUAD samples. **F** FRAS1 protein level was detected by western blot in five pairs of LUAD samples. **G** FRAS1 expression at mRNA level was analyzed by qRT-PCR in NSCLC cell lines including A549, HCC827, NCI-H1975, and NCI-H1299, and lung epithelial cell line HBEC. **H** The m6A level of FRAS1 was analyzed in the above five cell lines. **I** FRAS1 expression at protein level was detected by western blot in A549, HCC827, NCI-H1975 and NCI-H1299, and HBEC. **P < 0.01, ***P < 0.001 indicates a significant difference between the indicated groups. NS, not significant

We used TCGA-LUAD/LUSC database to analyze the correlated genes with METTL3 and YTHDF1, respectively. FRAS1 was identified to be the gene most associated both with METTL3 and YTHDF1 in non-small cell lung cancer (NSCLC) as shown in Fig. 1A (R_METTL3_ = 0.730, R_YTHDF1_ = 0.452, *P* < 0.001, Additional file 1: Table S1). Next, we found an interesting phenotype that the mRNA level of FRAS1 was significantly lower in 106 paired of NSCLC tumor tissues than that in the corresponding normal tissues (Fig. 1B) whereas the Kaplan–Meier plotter analysis indicated that high FRAS1 expression was associated with shorter overall survival of NSCLC patients (log rank *P* = 7e-04, https://kmplot.com/analysis/, Fig. 1C), suggesting that FRAS1 might be a risk prognosis biomarker in NSCLC. We wondered whether there was m6A modification of *FRAS1* mRNA in lung cancer. Then, we evaluated the expression of *FRAS1* and its m6A level in our collected LUAD samples. Consistent with the public database analysis, *FRAS1* mRNA level was decreased in tumor tissues compared to normal tissues (Fig. 1D). However, the m6a level and the protein level of *FRAS1* was significantly increased in 3 cases of tumor tissues compared to the paired corresponding normal tissues (Fig. 1E, F). A similar result was also observed in NSCLC cell lines. *FRAS1* mRNA level was decreased in NSCLC cell lines A549, HCC827, NCI-H1975 and NCI-H1299 compared to the human bronchial epithelial cells HBEC (Fig. 1G). The m6A level and protein level of *FRAS1* in NSCLC cell lines were higher than in HBEC (Fig. 1H, I). These results suggest that m6A modification of *FRAS1* mRNA and its protein level was obviously increased in NSCLC tumor tissues.

### METTL3 induces *FRAS1* mRNA m6A modification

**Fig. 2:**
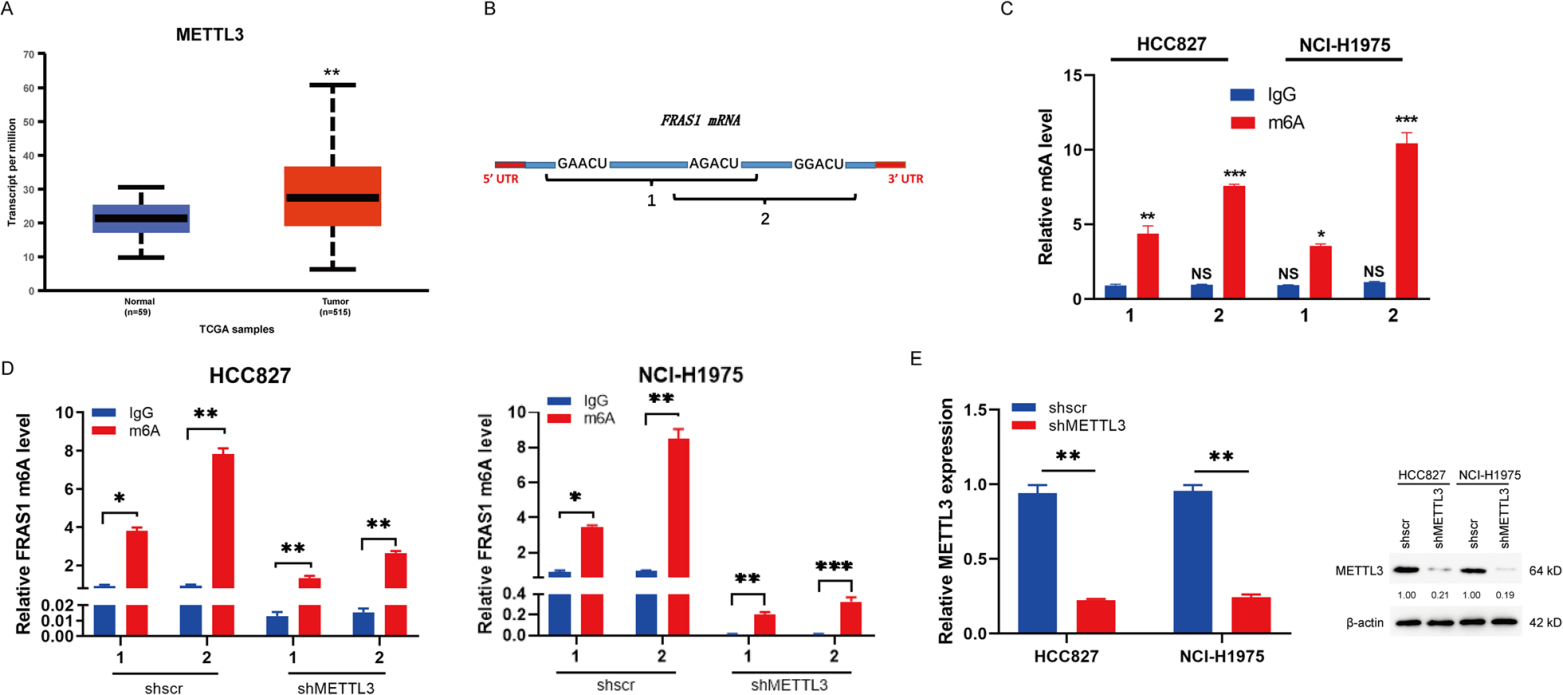
The FRAS1 m6A methylation site occurs near the terminator codon in NSCLC. **A** The mRNA level of METTL3 in NSCLC was analyzed using UALCAN (http://ualcan.path.uab.edu/). **B** The predicted m6A motifs in the coding sequence (CDS) of FRAS1. Two PCR primers designed for different FRAS1 DNA segments, as marked by PCR 1 and PCR 2. **C** FRAS1 DNA fragments were amplified by qRT-PCR after m6A immunoprecipitation using m6A antibody in HCC827 and H1975 cells, which was normalized to IgG control of HCC827. **D** The m6A level was detected in METTL3-knockdown HCC827 and NCI-H1975 cells by qRT-PCR after m6A immunoprecipitation. **E** The knockdown efficiency of shMETTL3 was evaluated by qRT-PCR and western blot. Values are represented as means ± SD. *P < 0.05 or **P < 0.01 or ***P < 0.001 indicates a significant difference between the indicated groups. NS, not significant

Emerging studies show that m6A plays a critical role in messenger RNA (mRNA) by regulating mRNA process, translation or decay [3]. For example, m6A may accelerate the processing time of mRNA precursors and promote mRNA transport and nucleation speed in cells [29, 30]. To investigate the regulatory mechanism of the m6A modification on *FRAS1* mRNA, we focused on the m6A “writer” METTL3. We observed that METTL3 was highly expressed in NSCLC tumor tissues compared to normal tissues (Fig. 2A). The m6A modification sites of *FRAS1* mRNA were predicted by SRAMP (http://www.cuilab.cn/sramp/) [31], of which the consensus motif were near the terminator (Fig. 2B). To verify the presence of *FRAS1* mRNA m6A methylation, we performed the m6A immunoprecipitation using m6A antibody. The immunoprecipitated RNA fragments were detected by qRT-PCR as shown in Fig. 2C and 2D. The m6A modification of *FRAS1* mRNA was both detected in HCC827 and NCI-H1975 cells (Fig. 2C). Moreover, the m6A methylation level in shMETTL3 cells (HCC827 and NCI-H1975) was remarkably reduced than the shscr group (Fig. 2D). There was 80% knockdown efficiency of shMETLL3 which was validated by qPCR and western blot (Fig. 2E). Above all, these results suggest that METTL3 could promote *FRAS1* transcript m6A modification.

### METTL3 silence led to the decreased FRAS1 protein expression and cell proliferation

**Fig. 3:**
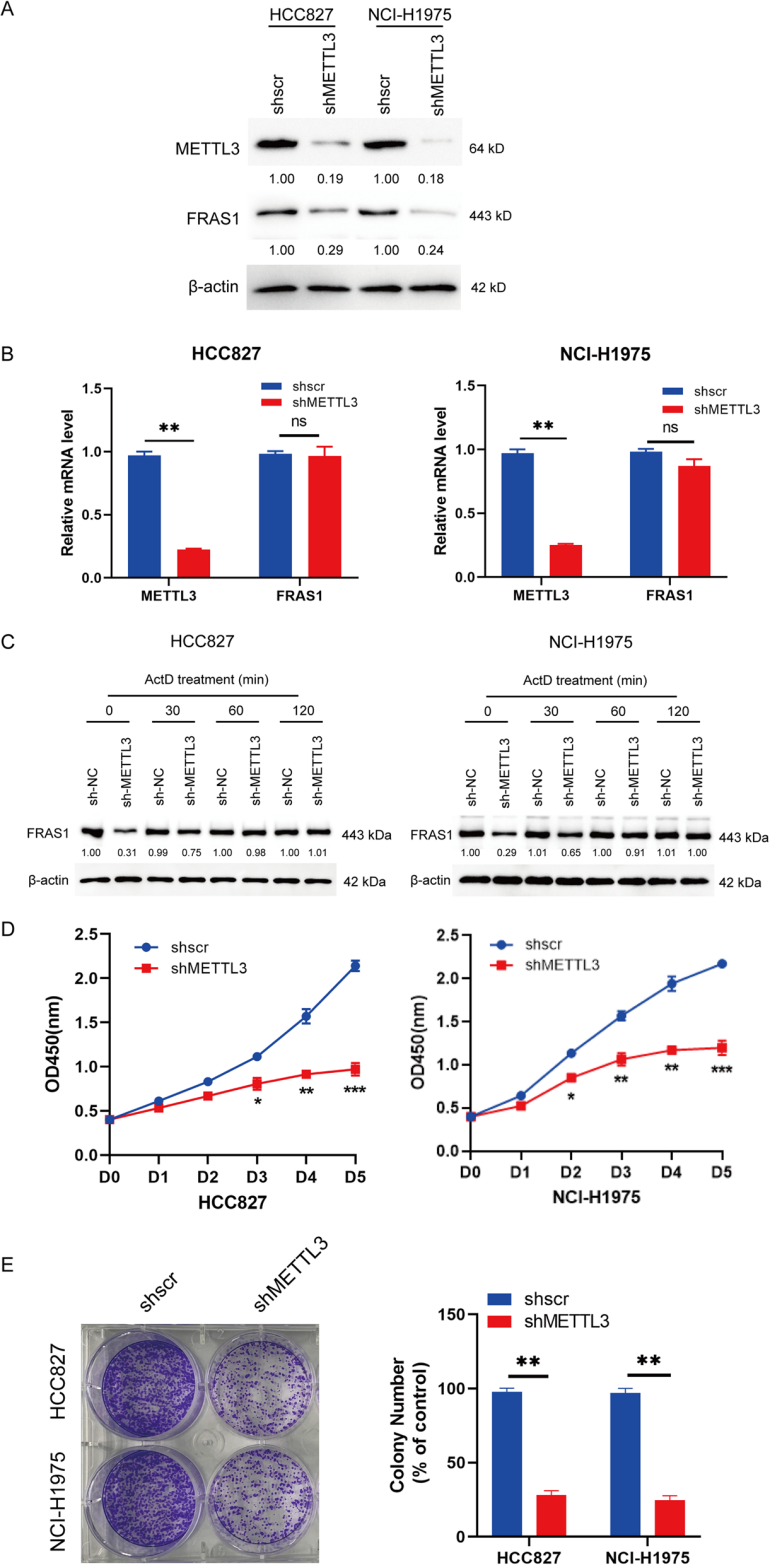
FRAS1 protein not mRNA level was decreased in METTL3-silenced cells. **A** The protein levels of METTL3 and FRAS1 were measured in METTL3-knockdown HCC827 and NCI-H1975 cells. **B** The mRNA level of METTL3 was determined in shMETTL3 HCC827 and NCI-H1975 cells by qPCR. **C** The protein level of FRAS1 was detected in METTL3-knockdown HCC827 and NCI-H1975 cells treated with ActD at 0, 30, 60, 120 min. **D** CCK-8 assay was performed in shMETTL3 HCC827 and NCI-H1975 cells. (**E**) Colony formation was performed in shMETTL3 HCC827 and NCI-H1975 cells for 4 days culture. *P < 0.05 or **P < 0.01 or ***P < 0.001 indicates a significant difference between the indicated groups. ns, not significant

To explore the biological function of METTL3-regulated *FRAS1* m6A modification, we generated the two stabilized METTL3-silenced cells (HCC827 and NCI-H1975). METTL3 was effectively decreased at protein level in shMETTL3 cells compared to shscr cells together with the reduced FRAS1 expression at protein level (Fig. 3A). There was no effect on the alteration of *FRAS1* mRNA upon METTL3 knockdown (Fig. 3B). Additionally, the protein level of FRAS1 was no longer reduced with the extension time of actinomycin D (ActD) treatment, suggesting that METTL3 regulated FRAS1 expression dependent on the m6A manner but had no direct effect on FRAS1 protein (Fig. 3C). CCK-8 analysis showed that cell proliferation was decreased in stable METTL3-silenced HCC827 and NCI-H1975 cells (Fig. 3D), which was consistent with the colony formation results (Fig. 3E). These results suggest that METTL3 alters FRAS1 protein level but not mRNA level and has an inhibiting action on cell proliferation.

### YTHDF1 plays a role in m6A-modified FRAS1 expression

**Fig. 4:**
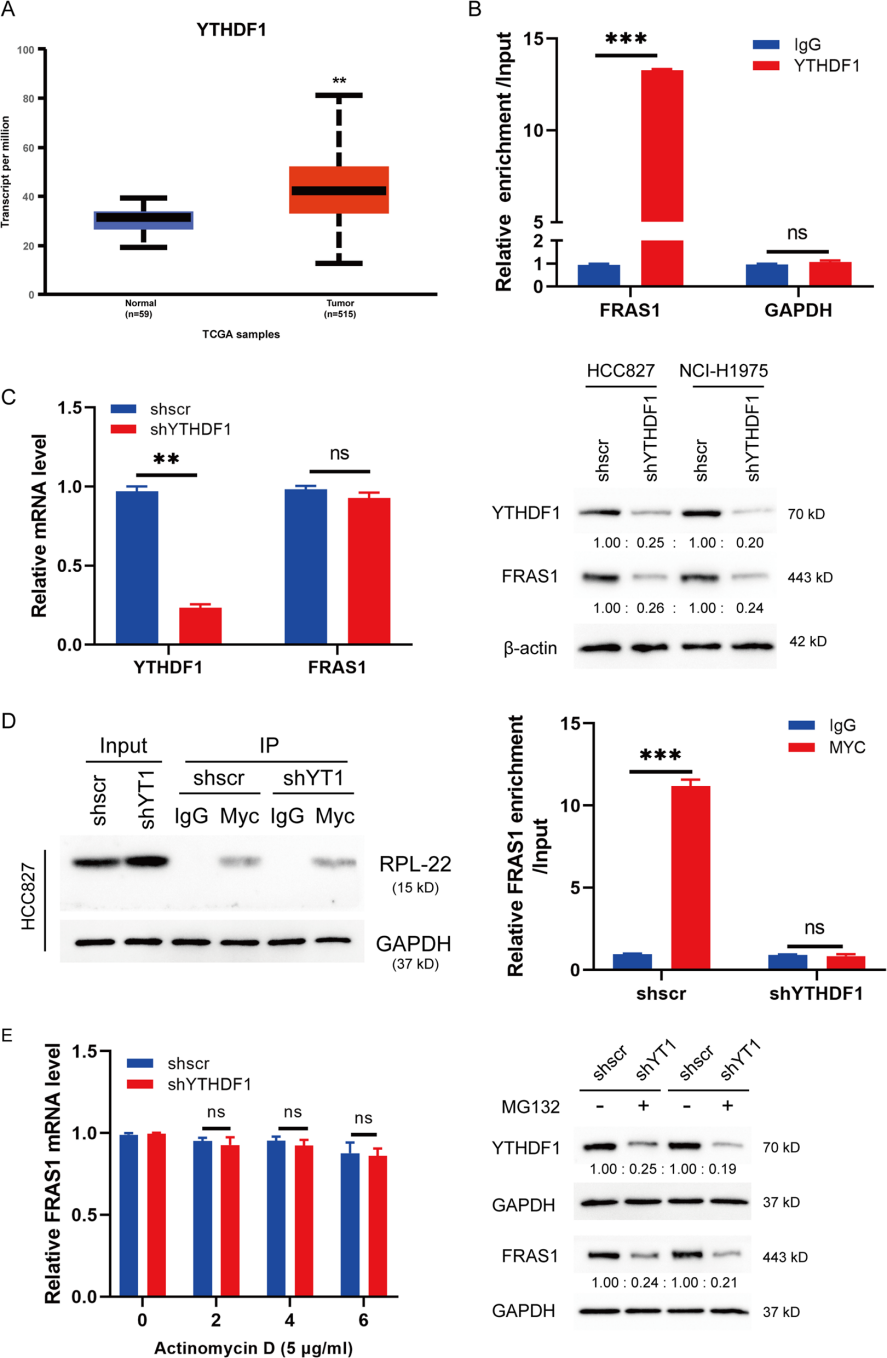
YTHDF1 keeps FRAS1 mRNA stability. **A** The mRNA level of YTHDF1 was analyzed in NSCLC via UALCAN. **B** The FRAS1 enrichment in RIP assay was performed using YTHDF1 antibody and quantified by qPCR. The relative FRAS1 enrichment was normalized to input. **C** The relative expression of YTHDF1 and FRAS1 at mRNA level was determined by qRT-PCR in HCC827 and NCI-H1975 cells with YTHDF1 silence (n = 3, left). **P < 0.01. The knockdown efficiency of shYTHDF1 was evaluated by western blot (right). **D** shYTHDF1 stable cells were transfected with plasmid Myc-RPL22 for 48 h. Ribosomal immunoprecipitation was performed using antibody Myc. The realtive FRAS1 expression was quantified by qPCR. **E** The mRNA level of FRAS1 was analyzed in shYTHDF1 stable cells with treatment of actinomycin D (5 μg/ml, left). The protein level of FRAS1 was detected in shYTHDF1 cells with or without MG132 (1 μM) for 24 h. GAPDH was used as an internal control. Values are represented as means ± SD. *P < 0.05 or **P < 0.01 or ***P < 0.001 indicates a significant difference between the indicated groups. ns, not significant

M6A modified RNAs could be recognized and transmitted to the downstream pathway by the m6A reader proteins. YTHDF1 has been identified to be critical for NSCLC proliferation [21] In this study, we found that YTHDF1 was highly expressed in NSCLC tumor tissues compared to normal tissues via UALCAN (Fig. 4A), indicating that YTHDF1 may play a critical role in m6A-regulated FRAS1 expression. The RIP assay validated that YTHDF1 could bind to *FRAS1* mRNA (Fig. 4B). To investigate the role of YTHDF1 in the regulation of m6A-modified *FRAS1*, we constructed stable YTHDF1-silenced HCC827 and NCI-H1975 cells. qRT-PCR analysis showed that YTHDF1 was significantly knockdown in shYTHDF1 cells, while *FRAS1* mRNA has no obvious change, but a remarkable decrease at protein level (Fig. 4C). Furthermore, we performed ribosome immunoprecipitation to analyze the YTHDF1-silence effects on the ribosome occupancy on m6A modified *FRAS1* mRNAs. Results indicated that the ribosome occupancy on *FRAS1* mRNA was reduced in YTHDF1-silenced cells compared to control cells (Fig. 4D). As expected, *FRAS1* mRNA stability was not changed when treated with ActD upon loss of YTHDF1, suggesting that YTHDF1 was not involved in *FRAS1* RNA synthesis. Next, to investigate whether YTHDF1 was involved in the regulation of FRAS1 protein stability, we treated cells with the proteasome inhibitor MG132. We observed that FRAS1 protein was still decreased in YTHDF1-silenced cells even with MG132 treatment (Fig. 4E), indicating that YTHDF1 could modulate FRAS1 protein stability not dependent on the protein degradation pathway. These results further suggest that m6A reader protein YTHDF1 may recognize the m6A modification of FRAS1, which was increased by METTL3.

### METTL3 induced FRAS1 m6A modification modulates CDON expression

**Fig. 5:**
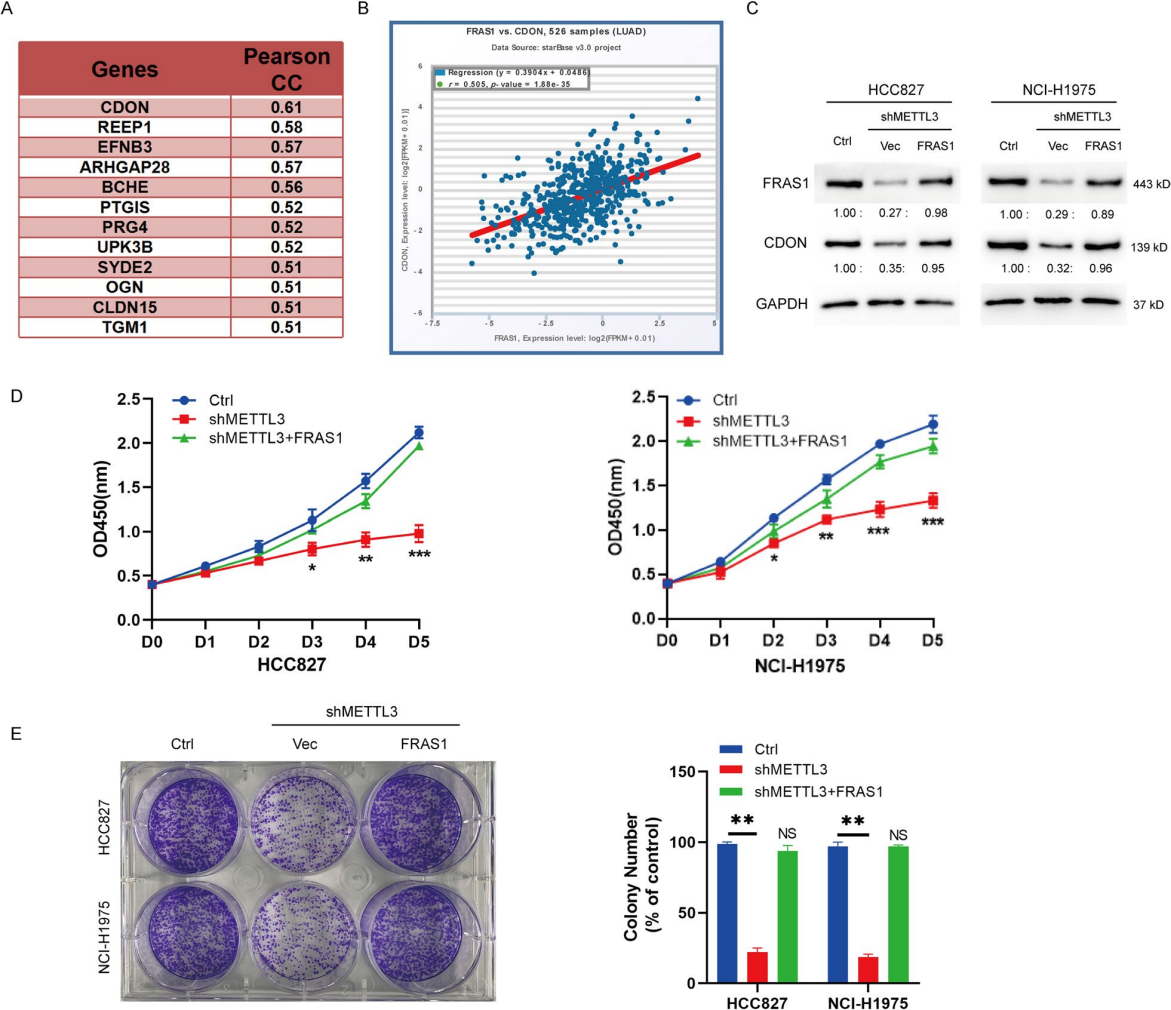
FRAS1 overexpression rescues the phenotype induced by METTL3 silence and CDON expression. **A** The positively associated genes with FRAS1 in NSCLC (pearson correlation r > 0.5). **B** The relationship between CDON and FRAS1 in NSCLC was analyzed using starbase (http://starbase.sysu.edu.cn/index.php). r = 0.505. *P* < 0.001. **C** The protein level of FRAS1 and CDON was analyzed by western blot in shMETTL3 HCC827 and NCI-H1975 cells with or without FRAS1 overexpression, or Ctrl cells. **D** CCK-8 assay was performed in shMETTL3 HCC827 and NCI-H1975 cells with or without FRAS1 overexpression, or Ctrl cells. *P < 0.05. **P < 0.01, ***P < 0.001. **E** Colony formation was analyzed in shMETTL3 HCC827 and NCI-H1975 cells with or without FRAS1 overexpression, or Ctrl cells

We found that cell adhesion associated, oncogene regulated (CDON) had a strong positive correlation with FRAS1 by the online bioinformatics analysis. The pearson analysis showed that CDON was the strongest correlated factor with FRAS1 in NSCLC (R = 0.61, Fig. 5A). Starbase online database revealed that there was a strong associated between FRAS1 and CDON in 526 pairs of NSCLC samples (r = 0.505, *P* < 0.001, Fig. 5B). In addition, FRAS1 overexpression could rescue the CDON protein level in shMETTL3 cells (Fig. 5C). CCK-8 analysis and colony formation analysis revealed that the decreased proliferation caused by METTL3 knockdown could be reversed by FRAS1 overexpression (Fig. 5D, E). These data suggest that METTL3 regulates cell proliferation via FRAS1.

In addition, we also preliminarily explored the biological role of CDON in HCC827 and NCI-H1975 cells by performing loss-of function by introducing siRNA targeting CDON. CDON expression at mRNA and protein level was significantly decreased in si-CDON-transfected cells as shown in Additional file 1: Figs. S1A and 1B. CCK-8 assay results showed that CDON downregulation led to reduced cell proliferation (Additional file 1: Fig. S1C). Consistently, we also observed that EDU positive cells were decreased when CDON was knockdown by performing EDU incorporation (Additional file 1: Fig. S1D). These results suggest that CDON may act as an oncogene in HCC827 and NCI-H1975 cells.

### FRAS1 is a target of N6-methyladenosine METTL3 in NSCLC

**Fig. 6:**
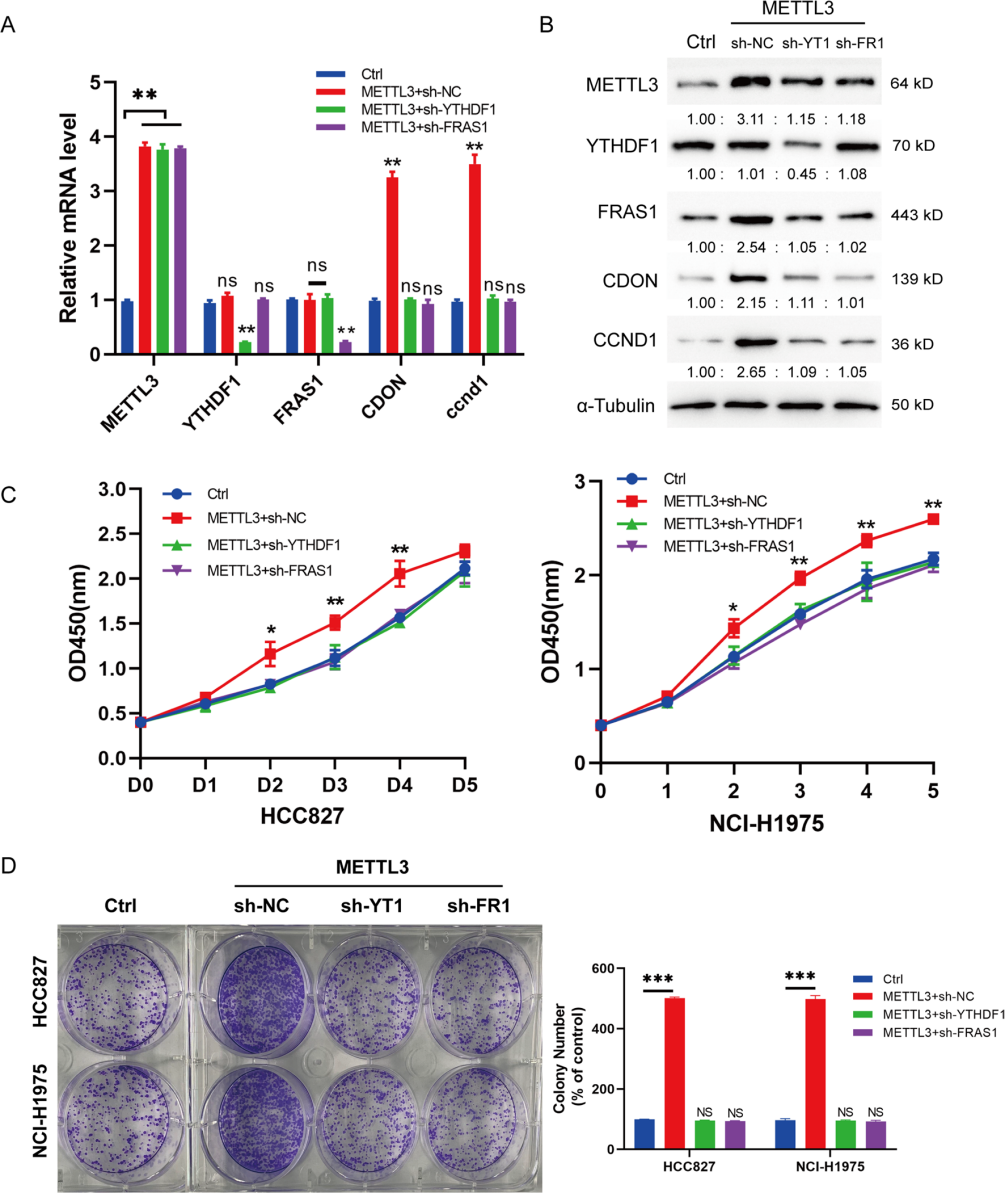
The enhanced FRAS1 m6A modification induced by METTL3 promotes cell proliferation of NSCLC cells. **A** The associated genes FRAS1, YTHDF1, CDON at mRNA level was analyzed in the following groups of HCC827 cells by qPCR. Ctrl, shscr + METTL3, shYTHDF1 + METTL3, shFRAS1 + METTL3. **B** The protein level of the above genes was measured by western blot in the following groups of HCC827 cells. Ctrl, shscr + METTL3, shYTHDF1 + METTL3, shFRAS1 + METTL3. **C** CCK-8 assay was analyzed in the following groups of HCC827 cells. Values are represented as means ± SD. (**D**) Colony formation was analyzed in the following groups of HCC827 cells. Ctrl, shscr + METTL3, shYTHDF1 + METTL3, shFRAS1 + METTL3. *P < 0.05 or **P < 0.01 or ***P < 0.001 indicates a significant difference between the indicated groups. ns, not significant

To further explore METTL3-induced *FRAS1* m6A modification in NSCLC cell proliferation, rescue experiments were performed in stable YTHDF1-silence, FRAS1-silence cells by overexpressing METTL3. qRT-PCR results showed that there was no obvious alteration on the mRNA level of *FRAS1* in METTL3-overexpression group compared to sh-YTHDF1 + METTL3-overexpression group (Fig. 6A). The upregulated FRAS1 and CDON protein levels induced by METTL3-overexpression could be restored by YTHDF1 silence or FRAS1 silence (Fig. 6B). Moreover, we also found that the elevated expression of cyclin D1 (CCND1) induced by METTL3 overexpression could be reversed by YTHDF1 knockdown or FRAS1 knockdown. *CCND1* which belongs to the highly conserved cyclin family, regulates cell cycle G1/S transition and promotes cell proliferation in multiple tumors [32–34]. Consistently, we also detected CDON, METTL3 and YTHDF1 protein expressions in five collected tumor samples as shown in Additional file 1: Fig. S2. The result showed that CDON expression was upregulated in T1, T2, T3 and T5 tumor tissues compared to their corresponding normal tissues but T4 sample. YTHDF1 and METTL3 expression at protein levels were all upregulated in five tumor tissues compared to their paired normal tissues. Functional studies showed that the METTL3-overexpression induced elevated cell proliferation could also be rescued by YTHDF1 silence or FRAS1 silence analyzed by CCK-8 and colony formation assay (Fig. 6C and D). Moreover, we also validated the expression of CDON, YTDHF1, and METTL3 in collected NSCLC tumor samples. It was observed that CDON, YTHDF1 and METTL3 were highly expressed in cancer tissues compared to that of in corresponding normal tissues (Additional file 1: Fig. S2), suggesting that there was a positive relationship between FRAS1, CDON, and METTL3/YTHDF1. Above all, our data indicate that FRAS1 was a major target of METTL3 regulating NSCLC cell proliferation.

### METTL3-induced FRAS1 promotes NSCLC tumor growth in vivo

**Fig. 7:**
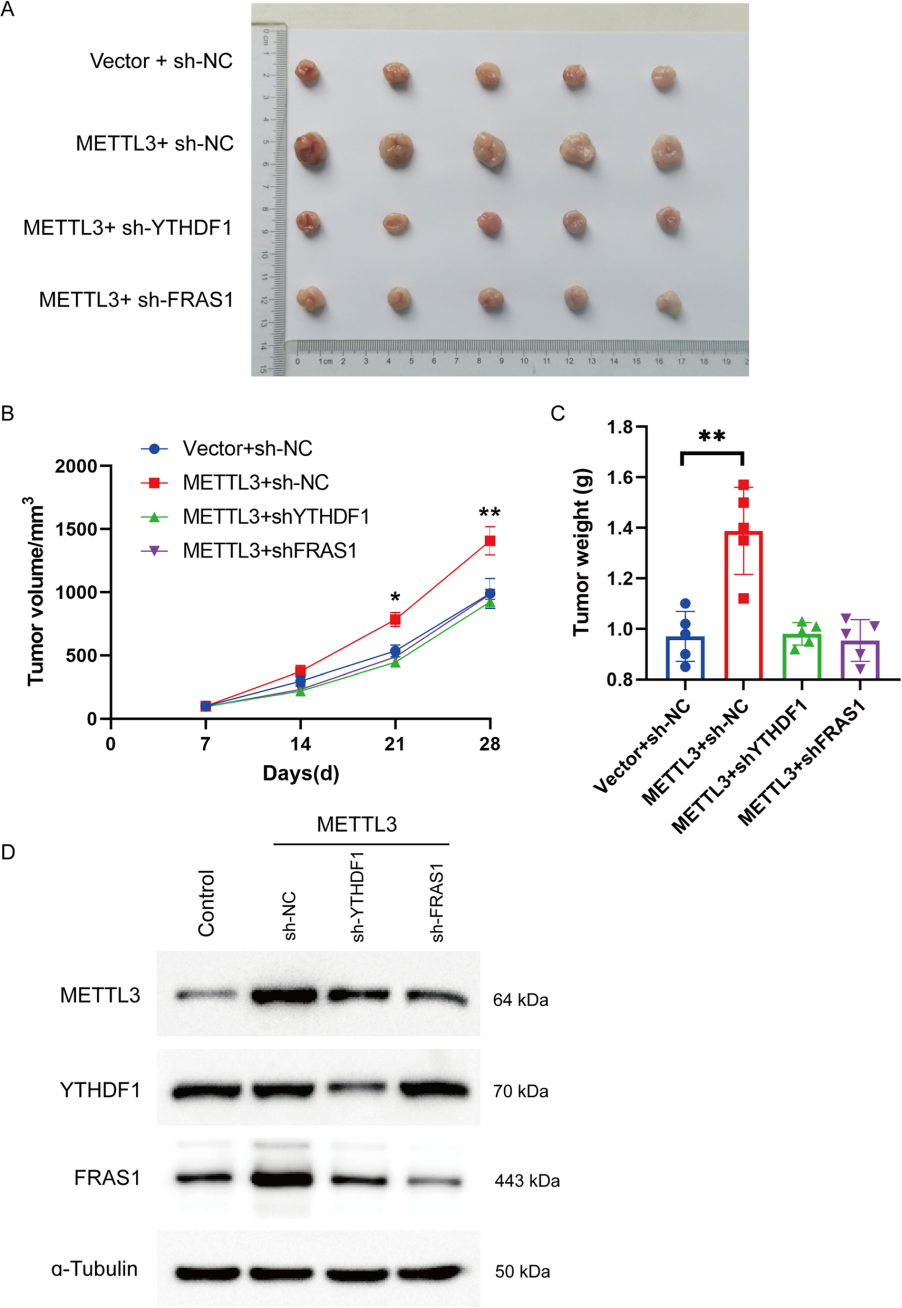
METTL3 induced FRAS1 promotes tumor growth in vivo. **A** Representative images of tumors originated from xenografted NCI-H1975 cells stably expressing Vector + sh-NC, METTL3 + sh-NC, METTL3 + sh YTHDF1, and METTL3 + sh-FRAS1 by subcutaneous injection. **B** The xenografte tumor model was constructed by using H1975 cells stably expressing Vector + sh-NC, METTL3 + sh-NC, METTL3 + sh YTHDF1, and METTL3 + sh-FRAS1 and the tumor volumn was measured at the indicated time points 7, 14, 21, 28 days after subcutaneous injection. **C** The tumor tissues were weighed after mice were sacrificed after 28 days. **D** The protein expression of METTL3, YTHDF1, and METTL3 was detected by western blot. *P < 0.05 or **P < 0.01 indicates a significant difference between the indicated groups

To confirm the role of METTL3/FRAS1 axis in vivo, we verified its effects on tumor growth using xenograft tumor model. As shown in Fig. 7, the excessive tumor growth induced by METTL3 upregulation was blocked by FRAS1 or YTHDF1 knockdown, including tumor volume and tumor weight (Fig. 7A–C). Consistently, the increased expression of FRAS1 caused by METTL3 overexpression was reduced when FRAS1 or YTHDF1 silence (Fig. 7D). Taken together, METTL3-induced FRAS1 promotes NSCLC tumor growth in vivo.

## Discussion

Lung cancer is considered to be the leading cause of cancer-related death in the world. The dysregulation of m6A modification is strongly associated with various tumor progression. The role of m6A regulators has been extensively reported in multiple tumors. However, not enough is known about the impact of m6A modification in lung cancer. This study addresses the role and functions of METTL3/YTHDF1/FRAS1 m6A in NSCLC cell proliferation.

In this study, we found an interesting phenomenon that there was an obvious difference between mRNA and protein level of FRAS1 in collected LUAD tissues, which was consistent in NSCLC cell lines. That is, there was no significant difference at mRNA level of *FRAS1* between NSCLC tumors and normal tissues whereas FRAS1 at protein level was upregulated in LUAD tumors compared to normal tissues. However, our results were not consistent with the TCGA analysis, of which the mRNA is lower in lung cancer tumor tissues than normal tissues. There were three possible reasons. The first reason is that the size of our collected sample was small, just five pairs of samples. We would collected more samples to identify the expression of *FRAS1* mRNA level between tumor tissues and normal tissues. Second, the tumor samples and normal samples were not paired in TCGA database. The difference in expression of *FRAS1* RNA level between tumor and normal tissues was not accurately reflected. Third, the lung cancer TCGA database includes data from small cell lung cancer (SCLC) as well as data from non-small cell lung cancer (NSCLC). Our collected samples were all NSCLC. The type of lung cancer might lead to the inconstant result between our study and the TCGA data analysis. In addition, the increased FRAS1 expression was related to the shorter overall survival of NSCLC patients. These results indicated that FRAS1 might be a tumor-promoter and a potential prognostic factor in NSCLC. FRAS1 was previously reported to be important in the process of epidermal-basement membrane adhesion during development [35]. Recent studies show that FRAS1 was associated with tumor metastasis [24, 25, 36]. FRAS1 was reported to facilitate the liver metastasis of gastric cancer through activating the EGFR and PI3K signaling pathways [24]. In NSCLC, it was found that FRAS1 knockdown could suppress HCC827 cell migration and invasion by inhibiting FAK signaling [23]. Our gain and loss-of functional studies revealed that FRAS1 overexpression could rescue decreased cell proliferation caused by METTL3 knockdown in HCC827 and NCI-H1975 cells. In turn, FRAS1 silence could reverse the elevated cell proliferation caused by METTL3 overexpression in NSCLC cells. This was the first to identify the role of FRAS1 on the promotion of cell proliferation in lung cancer.

We found that there were conserved methylation sites near the stop codon of *FRAS1* mRNA. METTL3, known as the key component of N6-methyltransferase complex, mediates tumorigenesis via RNA methylation [37]. In this work, we found that METTL3 was highly expressed in NSCLC tumors. Methylated RNA immunoprecipitation revealed that *FRAS1* mRNA existed m6A modification mediated by METTL3. METTL3 was reported to have oncogenic or tumor-suppressor roles in a series of tumors. Such as, METTL3 promotes liver cancer progression by regulating SOCS2 in an YTHDF2-dependent manner [9]. METTL3 promotes gastric cancer progression by regulating HDGF expression dependent on m (6)A modification [38]. In papillary thyroid cancer (PTC), METTL3 restrained PTC progression via m6A/c-Rel/IL-8-mediated neutrophil infiltration [39]. METTL3 has been identified to be a therapeutic target for lung cancer. YAP could promote the generation of lung cancer stem cells in a METTL3-m6A-YTHDF3-dependent manner [14], METTL3 could reverse gefitinib resistance of NSCLC by β-elemene [40]. Because of its importance in lung cancer, it is essential to investigate its detailed mechanisms. In this study, we observed that MELLT3 knockdown inhibited NSCLC cell lines HCC827 and NCI-H1975 cell proliferation and colony formation. It is the first study about the correlation between m6A modification of FRAS1 and lung cancer cell proliferation.

It is known that m6A modifiers could influence tumorigenesis in an m6A manner. For example, METTL3 could promote lung cancer tumor growth and invasion by boosting the translation of EGFR or TAZ transcripts independent of its catalytic activity [13]. In this study, we found that the elevated cell proliferation caused by METTL3 up-regulation could be restored by FRAS1 silence or YTHDF1 silence, indicating that METTL3 affected NSCLC cell proliferation in a m6A-depenent manner. However, it would be more complete to perform additional studies to identify whether a possible mutual effect of other writers such as METTL4 over FRAS1 mRNA is possible or if other readers such as YTHDF2 are involved.

M6A readers were reported to be responsible for the methylated mRNA fate. Our data first verified that YTHDF1 could promote the translation of m6A-modified *FRAS1* mRNA. These data were consistent with the previous research [41]. However, the consistency between cellular and tissue level expression of METTL3, YTHDF1 and CDON was not verified. Moreover, further biological function controlled by YTHDF1-promoted FRAS1 protein stability deserves detailed study.

The m6A modification is dynamic and reversible, which could be removed by “eraser” m6A demethylase enzymes, such as FTO, ALKBH5 and demethylases et al. It is reported that FTO could promote hepatocellular carcinoma tumorigenesis via triggering PKM2 demethylation [42]. A study reported that ALKBH5 could maintains tumorigenicity of glioblastoma stem-like cells by sustaining FOXM1 expression [43]. In this study, we mainly focused on FRAS1 m6A modification by “Writers” and “Readers”. It would be interesting to investigate the possible regulatory mechanisms of FRAS1 modification by erasers enzymes such as FTO, which will be performed in our further project and enrich the regulatory mechanism mediated by FRAS1 in NSCLC.

CDON (cell adhesion associated, oncogene regulated) is a cell surface protein that activates SHH signaling, which plays an important role in endothelium integrity [44, 45]. Nowadays, CDON was identified to participate in tumor progression. It was reported that CDON was involved in tumor cell growth and invasion in prostate cancer [41]. In this study, METTL3/YTHDF1 elevated FRAS1 protein expression, thus accelerating cell proliferation and tumor growth (colony formation). Meanwhile, we observed that FRAS1 modulated CDON expression. The increased expression of CDON in METTL3-overexpression cells was reversed by FRAS1 silence or YTHDF1 silence. These findings were consistent with the previous reports, that is, CDON was crucial for NSCLC cancer cell proliferation and tumorigenesis [41].

To sum up, our study first demonstrates the tumor-promoter role of FRAS1 in NSCLC and provides an m6A-dependent regulatory mechanism by METTL3. The m6A methyltransferase METTL3 promotes FRAS1 protein levels by enhancing FRAS1 mRNA m6A modification and FRAS1 transcript stability. The regulatory network of “writer” METTL3, “reader” YTHDF1, and “target” FRAS1 inspires the understanding of m6A-dependent gene regulatory mechanism in cancer biology, which offers a possibility of m6A regulators as promising biomarkers in NSCLC.

## Supplementary Information

**Additional file 1:** Supplementary figures and tables.

## Data Availability

The datasets generated during the current study are available from the corresponding author on reasonable request.

## Abbreviations

m6A: N6-methyladenosine
NSCLC: Non-small cell lung cancer
LUAD: Lung adenocarcinoma
FRAS1: Fraser extracellular matrix complex subunit 1
CDON: Cell adhesion associated, oncogene regulated
METTL3: Methyltransferase 3, N6-adenosine-methyltransferase complex catalytic subunit
YTHDF1: YTH N6-methyladenosine RNA binding protein 1
IP: Immunoprecipitation
RIP: RNA immunoprecipitation
qRT-PCR: Quantitative Reverse Transcription-Polymerase chain reaction
CDS: Coding sequence
HR: Hazard ratio
CI: Confidence Interval
PCR: Polymerase chain reaction
CCK-8: Cell Counting Kit
EDU: 5-Ethynyl-2’-deoxyuridine
