# Correction: METTL3 promotes non-small cell lung cancer (NSCLC) cell proliferation and colony formation in a m6A-YTHDF1 dependent way

**DOI:** 10.1186/s12890-022-02244-z

**Published:** 2022-11-28

**Authors:** Xuejun Dou, Zhiyuan Wang, Weiqiang Lu, Libin Miao, Yuefeng Zhao

**Affiliations:** Department of Thoracic Surgery, Aerospace Center Hospital, Beijing, China

## Correction to: BMC Pulmonary Medicine (2022) 22:324 https://doi.org/10.1186/s12890-022-02119-3

Following publication of the original article [1], the authors flagged an error in the affiliation information: 'Space Central Hospital' was detailed in place of 'Aerospace Center Hospital'. The affiliation has now been corrected in the published article.
