# Supplementary material for: METTL3 promotes non-small cell lung cancer (NSCLC) cell proliferation and colony formation in a m6A-YTHDF1 dependent way
Source: BMC Pulm Med. 2022 Aug 25;22:324. doi: 10.1186/s12890-022-02119-3 (PMC9413890; doi:10.1186/s12890-022-02119-3)
Supplement: Supplementary file 1 — Additional file 1: Supplementary figures and tables. [file 12890_2022_2119_MOESM1_ESM.docx]

**Supplementary Figure 1 CDON downregulation could reduce cell proliferation in HCC827 and NCI-H1975 cells.**


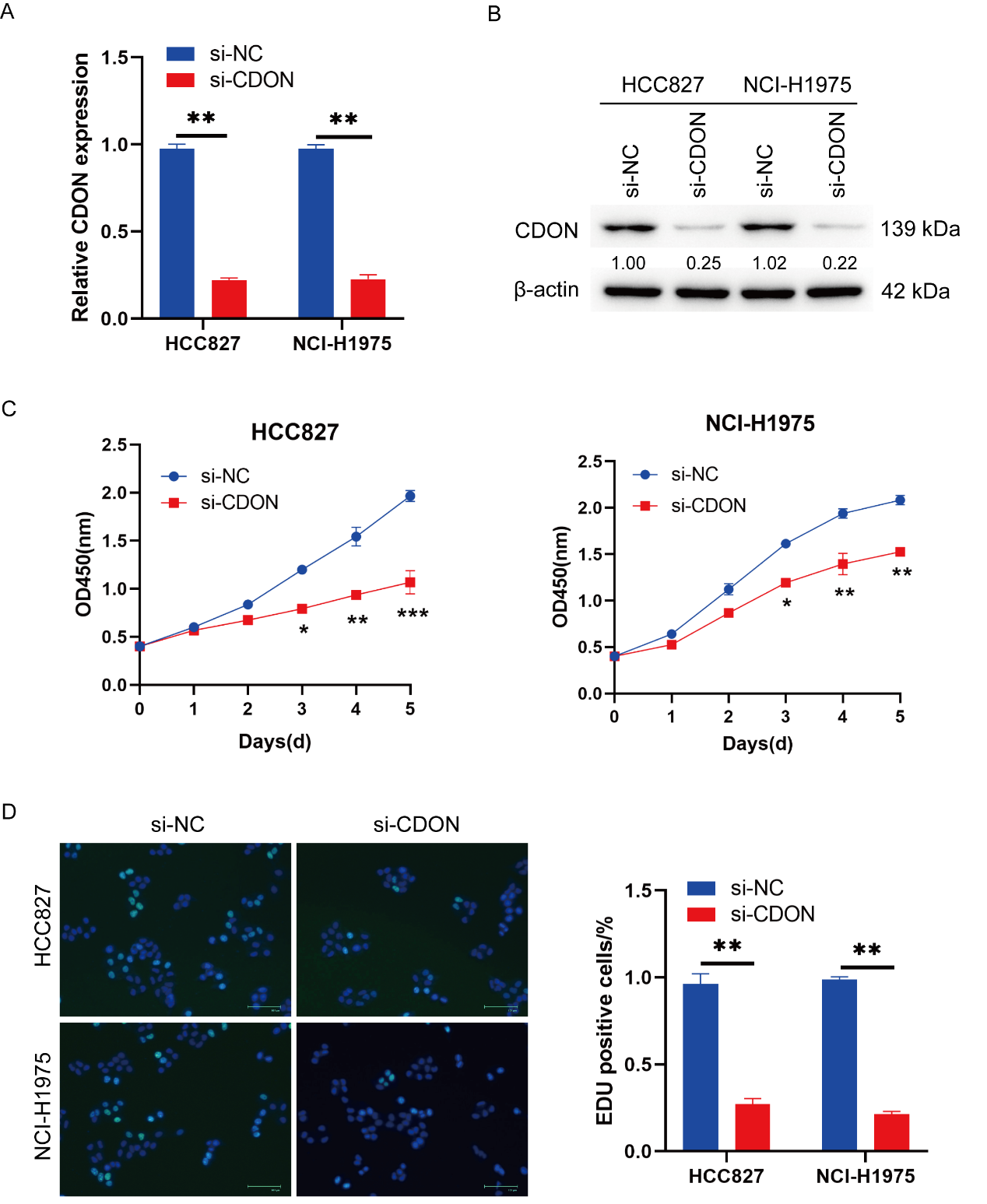


(A) CDON mRNA level was examined in si-CDON transfected HCC827 and NCI-H1975 cells by qPCR. (B) CDON protein level was detected in si-CDON transfected HCC827 and NCI-H1975 cells by western blot. (C) The cell viability was detected in si-CDON transfected HCC827 and NCI-H1975 cells. Values are represented as means ± SD. (D) EDU incorporation was performed in HCC827 and NCI-H1975 cells in si-CDON transfected cells. *P < 0.05 or **P < 0.01 or ***P <

**Supplementary Figure 2 CDON, METTL3 and YTHDF1 expressions at protein level were detected in five collected paired NSCLC tumor samples.**


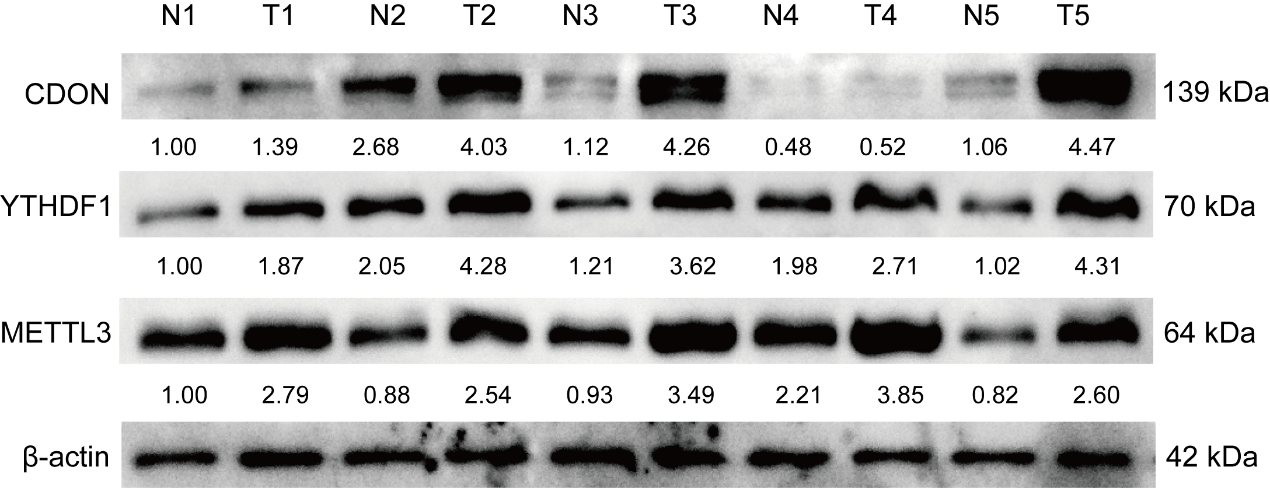


**Supplementary Table 1 Genes correlated with METTL3 and YTHDF1**

| **Only Genes co-related with METTL3** | **Only Genes co-related with YTHDF1** | **Genes co-related with METTL3 AND Genes co-related with YTHDF1** |
| --- | --- | --- |
| **TRUB1** | **SLC26A10** | **FRAS1** |
| **GJA8** | **LZTR1** | **PLA2G3** |
| **CHN1** | **KLC3** | **BTNL2** |
| **PDK3** | **PON1** | **RPAP3** |
| **SARM1** | **ERV3-1** | **MS4A2** |
| **KLF8** | **POU5F1B** | **TAGLN** |
| **MISP** | **BCAR1** |  |
| **SMARCD1** | **MICAL1** |  |
| **VPS13A** | **LIN54** |  |
| **SYTL3** | **HGD** |  |
| **ACTBL2** | **ELF3** |  |
| **LIN37** | **JADE2** |  |
| **HSD17B7** | **ADGRL3** |  |
| **TSPAN31** | **CR1L** |  |
| **COASY** | **EXOC1L** |  |
| **AP000275.2** | **SNX9** |  |
| **METTL13** | **ATG7** |  |
| **SNU13** | **OR8B8** |  |
| **FBXO2** | **FAU** |  |
| **DNAH1** | **TRAF3** |  |
| **NDFIP2** | **CD9** |  |
| **FAM114A2** | **UBR5** |  |
| **CHCHD1** | **CEP89** |  |
| **KLRD1** | **TERF2** |  |
| **ADAM20** | **SPDYE3** |  |
| **ANAPC4** | **KRTAP27-1** |  |
| **USP10** | **HLA-E** |  |
| **AKAP11** | **KDM7A** |  |
| **SLC22A1** | **CDH22** |  |
| **ATP8B4** | **PPM1F** |  |
| **FAM162A** | **IQCH** |  |
| **KATNAL1** | **PAEP** |  |
| **ALKAL2** | **CNBD2** |  |
| **TLX3** | **TPST2** |  |
| **CNKSR2** | **LTB4R2** |  |
| **HIRIP3** | **MFNG** |  |
| **F7** | **ZNF585B** |  |
| **ANXA8** | **GZMM** |  |
| **PIK3IP1** | **HIGD1B** |  |
| **PRSS23** | **AL662899.3** |  |
| **STK24** | **FAM186B** |  |
| **ZNF319** | **PRSS54** |  |
| **SLC36A2** |  |  |
| **DMC1** |  |  |
| **AJAP1** |  |  |
| **CD276** |  |  |
| **USB1** |  |  |
| **SRRD** |  |  |
| **TTC17** |  |  |
| **BOLA2-SMG1P6** |  |  |
| **C11orf91** |  |  |
| **FRRS1** |  |  |
| **OR5A1** |  |  |
| **KRR1** |  |  |
| **PSAT1** |  |  |
| **STARD7** |  |  |
| **TTC28** |  |  |
| **SERPINB1** |  |  |
| **C1orf27** |  |  |
| **EHMT2** |  |  |
| **WDR54** |  |  |
| **INTS6** |  |  |
| **RAI2** |  |  |
| **PPP1R2P3** |  |  |
| **INSL5** |  |  |
| **EIF3L** |  |  |
| **HAND1** |  |  |
| **FOXP2** |  |  |
| **MAPK4** |  |  |
| **MCU** |  |  |
| **MAPK7** |  |  |
| **CC2D2B** |  |  |
| **GNAI3** |  |  |
| **C6orf136** |  |  |
| **CXCL3** |  |  |
